# Next‐generation sequencing‐based IG clonality analysis to discriminate reactive infiltrates from minimal lymphoma involvement in paired lymphoma and bone marrow biopsies; a EuroClonality‐NGS Working Group study

**DOI:** 10.1111/his.70073

**Published:** 2025-12-15

**Authors:** Michiel van den Brand, Meilinde Leenders, Jos Rijntjes, Jeroen A.C.W. Luijks, Anton W. Langerak, Konnie M. Hebeda, Patricia J.T.A. Groenen

**Affiliations:** ^1^ Department of Pathology Radboud University Medical Center Nijmegen the Netherlands; ^2^ Laboratory Medical Immunology, Department of Immunology Erasmus MC Rotterdam The Netherlands

**Keywords:** B‐cell lymphoma, bone marrow, clonality analysis, immunoglobulin gene rearrangements, next‐generation sequencing, staging

## Abstract

**Aims:**

Bone marrow (BM) biopsy is an important procedure in B‐cell lymphoma staging. In most biopsies, the presence or absence of a lymphoma infiltrate can reliably be determined by standard histology. However, in a subset of cases with limited infiltration, this assessment remains inconclusive, requiring an alternative approach. Next‐generation sequencing (NGS)‐based detection of immunoglobulin (IG) gene rearrangements has the potential for resolving these difficult cases because of its high sensitivity. In this study, we tested the NGS‐based IG clonality protocol developed by the EuroClonality‐NGS Working Group on BM staging biopsies.

**Methods and results:**

Forty‐nine BM biopsies ranging from morphologically and immunohistochemically evidently involved to negative were analysed and compared to the original lymphoma. A clear distinction in the abundance of overlapping clonal IG rearrangements was observed between BM biopsies that were positive versus negative for lymphoma based on morphology and immunohistochemistry. In the 12 BM biopsies in which morphology and immunohistochemistry were insufficient to differentiate between the presence or absence of lymphoma, the estimated B‐cell infiltration ranged from 1% to 5%. In these cases, NGS‐based IG clonality analysis of paired primary lymphoma/BM biopsies provided a binary outcome; a subset of cases with hardly or no primary lymphoma‐derived IG gene rearrangements in the BM biopsy could be distinguished from cases with clear presence of primary lymphoma‐derived IG gene rearrangements.

**Conclusions:**

Our data demonstrated that paired NGS‐based IG clonality analysis of lymphoma and BM samples can be a valuable additional tool for difficult BM staging biopsies in patients with B‐cell lymphoma.

## Graphical abstract

Next‐generation sequencing (NGS) immunoglobulin clonality analysis can help resolve ambiguous bone marrow staging biopsies for B‐cell lymphoma. In this study, paired analysis of the primary lymphoma and bone marrow helped to detect lymphoma involvement in cases with inconclusive morphology and immunohistochemistry, making it a valuable tool for difficult staging biopsies.

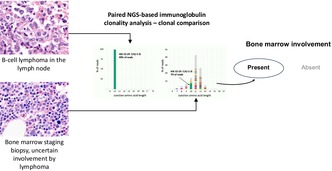

**Abbreviations**
BM: bone marrow
CT: computed tomography
DLBCL: diffuse large B‐cell lymphoma
FDG: fluorodeoxyglucose
FFPE: formalin‐fixed paraffin‐embedded
FL: follicular lymphoma
IG: immunoglobulin
IGH: IG heavy chain
IGK: IG kappa
MCL: mantle cell lymphoma
MZL: marginal zone lymphomas
NGS: next‐generation sequencing
PET: positron emission tomography

## Introduction

Evaluation of a bone marrow (BM) biopsy is an important component of the staging for most types of B‐cell lymphoma.^1^, ^2^ Although a BM biopsy is no longer routinely included in the staging of aggressive B‐cell lymphoma or Hodgkin lymphoma due to improvements in fluorodeoxyglucose (FDG) positron emission tomography (PET)‐computed tomography (CT) imaging techniques, BM biopsy remains important in the staging of low‐grade B‐cell lymphoma and in a small subset of aggressive B‐cell lymphomas without bone marrow involvement by FDG‐PET/CT.^3^, ^4^ If the biopsy is of sufficient quality and length, a conclusion on the presence or absence of BM involvement can usually be made with confidence on the basis of standard immunohistochemistry and morphology. To evaluate a BM biopsy for lymphoma infiltration, the first step is to evaluate the number and distribution of B cells. A significant increase in the number of B cells compared to normal BM is suggestive of lymphoma involvement in the absence of autoimmune disease, infection or previous chemotherapy. Next, evaluation of the pattern of involvement is important; paratrabecular, intrasinusoidal or diffuse infiltration of B‐cell aggregates is abnormal, whereas a nodular or interstitial increase in B cells could still be reactive. In difficult cases, the morphology of the B cells can be helpful; sheets of blasts or very monotonous lymphoid proliferations favour lymphoma involvement. An abnormal immunohistochemical pattern can also help to confirm the presence of lymphoma, for example expression of cyclin D1 in mantle cell lymphoma, CD5 expression in chronic lymphocytic leukaemia or mantle cell lymphoma or light chain restriction in lymphomas with plasmacytic differentiation.^5^ Nevertheless, in a small subset of BM staging biopsies a final conclusion cannot be made because a differential diagnosis between subtle BM involvement and the presence of a reactive lymphoid proliferation remains, even after careful consideration of morphology and immunohistochemistry.^6^, ^7^

Clonality analysis has added value to resolve a subset of these ambiguous staging biopsies. Conventional immunoglobulin (IG) clonality analysis with PCR amplification followed by fragment size analysis (GeneScan) of IG heavy chain (IGH) and IG kappa (IGK) genes has been performed previously and detection of a clonal product in the BM biopsy was shown to have an impact on prognosis in patients with follicular lymphoma (FL) and diffuse large B‐cell lymphoma (DLBCL).^8^, ^9^ Also next‐generation sequencing (NGS)‐based IG clonality assays have been used as ancillary tests to detect BM involvement in patients with B‐cell lymphoma.^10^, ^11^

In this study, we used a recently developed NGS‐based IG clonality analysis protocol^12^, ^13^, ^14^ to detect BM involvement in patients with lymphoma, irrespective of the type of B‐cell lymphoma. Our NGS‐based IG clonality approach has been optimized for material with suboptimal DNA quality, in contrast to IGH FR1 and IGK assays used in previous studies.^10^, ^11^ It has been tested on a large series of both reactive and neoplastic lymphoproliferations in which it showed high sensitivity and specificity.^13^ In addition, guidelines for interpretation of the results using an algorithmic approach have been published.^15^ A great advantage of using an NGS‐based IG clonality assay is that it allows very accurate clonal comparison between the material of the primary lesion and the BM, because the sequence of the IGH and/or IGK gene rearrangements of the lymphoma clone is used for comparison rather than the fragment size of the PCR products using the conventional methodology. The IG sequences are ascribed as clonotypes that are characterized by the same V and J gene and the junctional region.^16^, ^17^ Similar to conventional clonality analysis, the NGS‐based approach uses multiple multiplex PCR reactions targeting both IGH and IGK, to increase chances of detecting the clones and to compensate for the possibility that a clone is not detected due to mutations in the primer binding sites caused by somatic hypermutation.

In this study, we analysed cases with or without BM involvement based on morphology and standard immunohistochemistry and cases with equivocal BM involvement. We show that NGS‐based IG clonality analysis can be an important additional diagnostic tool in BM staging biopsies with ambiguous lymphoma involvement.

## Materials and Methods

### Patient Selection

Material was retrieved from the archive of the Department of Pathology, Radboud University Medical Center, the Netherlands. In total, 49 BM trephine biopsies from 2018 were included from four categories: (1) patients with a B‐cell lymphoma having a BM biopsy that was positive for lymphoma based on morphology and immunohistochemistry (n = 11; 7 FL, 3 mantle cell lymphoma (MCL) and 1 case of chronic lymphocytic leukaemia/small lymphocytic lymphoma); (2) patients with a B‐cell lymphoma having a BM biopsy that was negative for lymphoma based on morphology and immunohistochemistry (n = 13; 8 DLBCL, 2 FL, 2 marginal zone lymphomas (MZL) and 1 Burkitt lymphoma (BL)); (3) patients with a B‐cell lymphoma in which the BM biopsy was equivocal for lymphoma involvement (n = 12; 4 DLBCL, 3 MZL, 2 FL, 2 MCL, 1 BL); and (4) BM biopsies from patients without lymphoma (n = 13).

For the patients with lymphoma, representative diagnostic material was used for comparison.

The study was conducted in accordance with the Declaration of Helsinki and the Declaration of Taipei and was performed on anonymized tissue remaining from routine care.

### Next‐Generation Sequencing IG Clonality Analysis

BM biopsies were received in Burckhardt's fixative and processed with a rapid fixation, decalcification, and paraffin impregnation procedure as published previously.^18^ Genomic DNA was isolated from formalin‐fixed paraffin‐embedded (FFPE) tissue by using 5% Chelex‐100 (Biorad, Hercules, CA) and proteinase K, followed by purification with the QIAamp DNA micro Kit (Qiagen, Venlo, the Netherlands). For a subset of the material on which the diagnosis of lymphoma was made, genomic DNA was isolated from fresh frozen material by lysis in TSE buffer, 20% SDS and proteinase K, followed by NaCl and ethanol precipitation. DNA concentrations were measured with a Qubit fluorometer (Invitrogen, Carlsbad, CA). DNA quality was assessed with a size ladder PCR as reported previously.^19^

Library preparation and high‐throughput sequencing were performed as described previously.^12^, ^13^ In short, EuroClonality‐NGS primer sets were used to amplify genes of the IG heavy chain (IGH) and IG kappa light chain (IGK) loci in three tubes, targeting four different rearrangement types: (1) IGHV‐IGHD‐IGHJ‐FR3; (2) IGHD‐IGHJ; (3) IGKV‐IGKJ and (4) IGKV‐KDE/IntronRSS‐KDE. After adapter ligation and purification, the libraries were sequenced on an IonTorrent platform (Thermo Fisher Scientific, Waltham, CA) using an IonTorrent 318 chip. Sequencing data were analysed with the ARResT/Interrogate platform (version 1.20.253).^20^ Targets IGH‐VJ‐FR3 and IGK‐VJ require at least 1000 reads to be assessable, whereas 500 reads is sufficient for targets IGH‐DJ, Vκ‐Kde and intron‐Kde (IGK‐Kde). The presence of lymphoma in the BM was measured as the ‘clonal overlap’. This was determined by first identifying the clonal rearrangements in the diagnostic lymphoma biopsy and then determining the percentage of reads in the BM staging biopsy corresponding to this clonal rearrangement. This percentage of lymphoma clonotypes that could be detected in the BM was reported as the clonal overlap.

## Results

### NGS‐Based IG Clonality Assessment Confirms Morphological Lymphoma Infiltration

For patients with a diagnosis of lymphoma, the BM biopsy and the corresponding lymphoma were assessed by NGS‐based IG clonality analysis for comparison. The results of these measurements are described in Tables 1 and 2.

**Table 1:** NGS‐based IG clonality analysis results for n = 11 cases positive for bone marrow involvement (group 1)

Case no.	Lymphoma/bone marrow	DNA quality (bp)	B‐cell %	Neoplastic cell %^b^	NGS IG clonality analysis results							
					IGHV‐IGHJ FR3		IGHD‐IGHJ		IGKV‐IGKJ		IGKV‐KDE/IntronRSS‐KDE	
					Target score	Rearrangement(s): clonotype^c^	Target score	Rearrangement(s): clonotype^c^	Target score	Rearrangement(s): clonotype^c^	Target score	Rearrangement(s): clonotype^c^
4	MCL	300	70	80	C/C	*V5‐51–1/33/−6 J4 94%/198* *V4‐34‐3/23/−1 J5 1%/2.2*	C	*D5‐24‐4/8/−7 J4 50%/20*	C	*V3(D)‐20–5/6/−4 J1 91%/164*	NSP	*NA*
	BM	300	20	80	C/C	*V5‐51–1/33/−6 J4 55%/39* *V4‐34‐3/23/−1 J5 22%/16*	C + PCB	*D5‐24‐4/8/−7 J4 35%/11*	C	*V3(D)‐20–5/6/−4 J1 87%/194*	NSP	*NA*
5	MCL	300	70	100	C	*V3‐7‐1/41/−6 J4 98%/1447*	P	*NA*	C	*V3(D)‐20–2/2/−3 J4 98%/1161*	NSP	*NA*
	BM	300	5	80	P	*V3‐7‐1/41/−6 2.5%/1.5*	P	*NA*	C + PCB	*V3(D)‐20–2/2/−3 J4 10%/6.1*	P	*NA*
11	FL	300	70	70	C	*V3‐23(D)‐3/30/−6 J3 63%/16*	MP	*NA*	P	*NA*	C/C	*V3‐11‐0/0/−0 Kde 56%/101* *intron − 0/0/−2 Kde 41%/74*
	BM	300	20	70	C	*V3‐23(D)‐3/30/−6 J3 58%/27*	MP	*NA*	P	*NA*	C/C	*V3‐11‐0/0/−0 Kde 53%/178* *intron − 0/0/−2 Kde 43%/146*
15	FL	200	80	80	NSP	*NA*	MP	*NA*	MP	*NA*	C	*V4–1‐1/0/−1 Kde 93%/40*
	BM	200	10	70	P	*NA*	P	*NA*	P	*NA*	C	*V4–1‐1/0/−1 Kde 91%/182*
17^a^	FL	200	50	70	C	*V3‐23(D)‐2/25/−6 J4 82%/21*	P	*NA*	Multiple products	*V2‐30‐7/2/−0 J4 31%/5.4* *V3D‐11–1/0/−4 J5 28%/4.9* *V2D‐29–0/0/−0 J1 28% 4.8*	C/C	*intron − 1/1/−13 Kde 56%/241* *intron − 0/0/−1 Kde 34%/145*
	BM	200	10	70	C	*V3‐23(D)‐2/25/−6 J4 55%/34*	P	*NA*	Multiple products	*V2‐30‐7/2/−0 J4 28%/5.4* *V3D‐11–1/0/−4 J5 25%/4.2* *V2D‐29–0/0/−0 J1 22%/3.8*	C/C	*intron − 1/1/−13 Kde 50%/60* *intron − 0/0/−1 Kde 32%/38*
31	FL	300	60	80	NSP	*NA*	NSP	*NA*	C	*V1(D)‐37–3/2/−7 J3 99%/1337*	C	*intron − 0/−4/−4 Kde 99%/1469*
	BM	300	20	80	MP	*NA*	MP	*NA*	C	*V1(D)‐37–3/2/−7 J3 97%/540*	C	*intron − 0/−4/−4 Kde 97%/478*
38	MCL	300	60	80	C	*V3‐53‐0/0/−7 J6 99%/196*	NSP	*NA*	C	*V3(D)‐20–3/4/−4 J1 97%/573*	NSP	*NA*
	BM	300	20	80	C	*V3‐53‐0/0/−7 J6 78%/158*	P	*NA*	C	*V3(D)‐20–3/4/−4 J1 63%/91*	P	*NA*
52	FL	300	60	80	P irr	*NA*	P irr	*NA*	C/C	*V4‐1‐3/0/−0 J1 41%/25* *V1(D)‐33–2/2/−6 J5 39%/24*	C + PCB	*intron − 0/0/−4 Kde 62%/39*
	BM	300	5	NA	P irr	*NA*	P irr	*NA*	C/C	*V4‐1‐3/0/−0 J1 17%/9.8* *V1(D)‐33–2/2/−6 J5 22%13*	C + PCB	*intron − 0/0/−4 Kde 63%/24*
53	CLL	200	70	90	C/C	*V3‐30 = V3‐33 = V3‐66‐0/29/−6 J4 70%/38* *V2‐26‐4/35/−6 J4 26%/14*	NSP	*NA*	C	*V2(D)‐30–1/0/−0 J1 98%/890*	C	*intron − 10/2/−1 Kde 98%/1039*
	BM	400	20	90	C/C	*V3‐30 = V3‐33 = V3‐66‐0/29/−6 J4 63%/48* *V2‐26‐4/35/−6 J4 24%/18*	P irr	*NA*	C	*V2(D)‐30–1/0/−0 J1 70%/329*	C	*intron − 10/2/−1 Kde 86%/308*
54	FL	300	70	80	P irr	*NA*	P irr	*NA*	P	*NA*	C/C	*V3‐11–1/0/−7 Kde 52%/319* *V3(D)‐20–1/0/−3 Kde 46%/282*
	BM	300	20	90	P irr	*NA*	P irr	*NA*	P	*NA*	C/C	*V3‐11–1/0/−7 Kde 54%/137* *V3(D)‐20–1/0/−3 Kde 33%/84*
55^a^	FL	300	80	90	C	*V1‐18‐4/17/−5 J5 95%/121*	NSP	*NA*	C/C	*V1‐27–1/0/−4 J5 59%/57* *V2‐24–1/0/−0 J5 40%/41*	C/C	*V1‐27‐3/0/−1 Kde 53%/103* *intron − 1/0/−8 Kde 43%/84*
	BM	400	70	90	C	*V1‐18‐4/17/−5 J5 93%/132*	NSP	*NA*	C/C	*V1‐27–1/0/−4 J5 52%/105* *V2‐24–1/0/−0 J5 47%/95*	C/C	*V1‐27‐3/0/−1 Kde 56%/72* *intron − 1/0/−8 Kde 40%/70*

For each case, NGS IG clonality analysis results are shown for the material on which the primary diagnosis of lymphoma was made and the corresponding bone marrow biopsy. When the BM was considered as clonal (C or C/C), the abundance percentage and ratio of the clonotypes are also provided. When the pattern is scored as polyclonal (irregular), but the index clonotypes of the lymphoma samples are represented in the polyclonal background, only the clonotype is noted in the table. No clonal overlap is reported when the index clonotype is not detected in the BM.

BM, bone marrow; bp, base pairs; C, clonal; CLL, chronic lymphocytic leukaemia; FL, follicular lymphoma; MCL, mantle cell lymphoma; MP, multiple products; NSP, no specific product; P, polyclonal; PCB, polyclonal background; P irr, polyclonal irregular; NA, not available.

^a^ The results for cases 17 and 55 have already been published in more detail.^14^

^b^ Expressed as a percentage of total B‐cells.

^c^ For the lymphoma cases, the clonal rearrangements are presented by their clonotype, the abundance percentage, and the ratio of the dominant #1 divided by an average measure of the background. When there is a dominance of two rearrangements, then also the abundance and ratio of the #2 clonotype are given.

**Table 2:** NGS‐based IG clonality analysis results for n = 13 cases negative for bone marrow involvement (group2)

Case no.	Lymphoma/bone marrow	DNA quality (bp)	B‐cell %	Neoplastic cell %^a^	NGS IG clonality analysis results							
					IGHV‐IGHJ FR3		IGHD‐IGHJ		IGKV‐IGKJ		IGKV‐KDE/IntronRSS‐KDE	
					Target score	Rearrangement(s): clonotype^b^	Target score	Rearrangement(s): clonotype^b^	Target score	Rearrangement(s): clonotype^b^	Target score	Rearrangement(s): clonotype^b^
16	MZL	200	50	80	NSP	*NA*	NSP	*NA*	MP	*NA*	C	*V1‐5‐2/0/−1 Kde 52%/9.2*
	BM	300	2	NA	MP	*NA*	MP	*NA*	P irr	*NA*	P irr	*No clonal overlap*
20	DLBCL	300	90	100	P	*NA*	P	*NA*	C/C	*V1(D)‐13–2/1/−0 J4 47%/42* *V2(D)‐28–3/0/−4 J5 12%/13*	C/C	*intron − 2/0/−3 Kde 42%/42* *intron − 3/0/−3 Kde 40%/41*
	BM	300	NA	NA	P irr	*NA*	P irr	*NA*	P	*No clonal overlap*	P	*0.97% intron − 2/0/−3 Kde*
26	DLBCL	300	90	90	C	*V3‐74–1/37/−4 J5 94%/184*	C	*D3‐3‐3/16/−9 J6 98%/1091*	C	*V4‐1‐13/12/−0 J2 97%/389*	C	*V1(D)‐13–1/0/−0 Kde 98%/1308*
	BM	200	NA	NA	P	*V3‐74–1/37/−4 J5 0.098%, minimal detection in polyclonal background*	P irr	*D3‐3‐3/16/−9 J6 0.82%*	P	*V4‐1‐13/12/−0 J2 0.098%*	NSP	*V1(D)‐13–1/0/−0 Kde 0.27%*
30	DLBCL	200	80	100	MP	*NA*	MP	*NA*	MP	*NA*	C/C	*V3‐11–1/0/−7 Kde 69%/565* *V3(D)‐20–1/0/−3 Kde 30%/243*
	BM	300	NA	NA	MP	*NA*	NSP	*NA*	MP	*NA*	P irr	*V3‐11–1/0/−7 Kde 0.29%*
36	MZL	400	50	80	C + PCB	*V4‐34‐0/27/−8 J5 18%/177*	C + PCB	*D2‐2‐10/31/−10 J6 12%/17*	C	*V1‐9‐1/2/−4 J4 47%/125*	P	*NA*
	BM	300	NA	NA	P	*No clonal overlap*	P	*No clonal overlap*	P	*No clonal overlap*	P	*NA*
41	DLBCL	300	80	100	C	*V3‐53‐2/27/−14 J6 98%/605*	C	*D3‐3‐4/0/−3 J5 99%/2680*	C	*V2(D)‐30–3/4/−0 J3 92%/482*	C	*V2(D)‐30–0/0/−1 Kde 96%/178*
	BM	300	2	NA	P irr	*0.012% V3‐53‐2/27/−14 J6*	P	*D3‐3‐4/0/−3 J5 0.019%*	P irr	*No clonal overlap*	P irr	*No clonal overlap*
42	BL	300	80	80	C/C	*V3‐30(−3) = V3‐64–1/45/−7 J4 53%* *V1‐69–1/15/−1 J6 37%*	NSP	*NA*	C	*V2‐29–0/1/−4 J2 96%/505*	C	*intron − 3/4/−4 Kde 96%/187*
	BM	300	10	NA	P	*V3‐30(−3) = V3‐64–1/45/−7 J4 1.9%* *V1‐69–1/15/−1 J6 0.47%*	P irr	*NA*	P	*V2‐29–0/1/−4 J2 2.3%*	P irr	*intron − 3/4/−4 Kde 2.0%*
43	DLBCL	300	80	100	C	*V4‐34‐5/10/−9 J2 96%/553*	NSP	*NA*	P irr	*NA*	P irr	*NA*
	BM	300	5	NA	P irr	*0.0018% V4‐34‐5/10/−9 J2*	P irr	*NA*	P	*NA*	P irr	*NA*
44	FL	300	60	80	C	*V4‐61–1/23/−6 J5 96%/178*	MP	*NA*	C/C	*V1(D)‐12–17/11/−2 J3 65%/61* *V3‐20 = V2D‐30‐3/0/−0 J4 27%/25*	NSP	*NA*
	BM	300	2	NA	P irr	*No clonal overlap*	P irr	*NA*	P	*No clonal overlap*	P irr	*NA*
45	DLBCL	300	80	100	C	*V4‐34‐0/30/−13 J6 88%/30*	NSP	*NA*	C	*V2‐29–1/0/−2 J4 99%/1218*	C	*intron − 3/6/−0 Kde 98%/836*
	BM	200	5	NA	P irr	*No clonal overlap*	P irr	*NA*	P irr	*No clonal overlap*	P irr	*No clonal overlap*
48	DLBCL	300	50	80	P irr	*NA*	C + PCB	*D2‐2‐6/2/−0 J4 52%/41*	C	*V1(D)‐33–1/0/−4 J4 74%/279*	C	*intron − 2/0/−3 Kde 80%/112*
	BM	400	10	NA	P	*NA*	P irr	*No clonal overlap*	P	*V1(D)‐33–1/0/−4 J4 0.27%*	P irr	*intron − 2/0/−3 Kde 1.1%*
49	DLBCL	300	80	80	C	*V3‐23(D)‐6/21/−7 J4 98%/534*	NSP	*NA*	C	*V3‐11‐0/0/−3 J4 99%/783*	C	*V2‐24‐4/2/−0 Kde 98%/688*
	BM	300	5	NA	P irr	*No clonal overlap*	P irr	*NA*	P	*V3‐11‐0/0/−3 J4 0.85%*	MP	*V2‐24‐4/2/−0 Kde 5.2%*
51	FL	300	60	80	P irr	*NA*	P irr	*NA*	C	*V1(D)‐13–1/1/−5 J4 84%/218*	C/C	*V2(D)‐30–0/0/−0 Kde 53%/104* *intron − 1/0/−4 Kde 39%/77*
	BM	300	5	NA	P	*NA*	P irr	*NA*	P	*V1(D)‐13–1/1/−5 J4 0.45%*	P irr	*No clonal overlap*

For each case, NGS IG clonality analysis results are shown for the material on which the primary diagnosis of lymphoma was made and the corresponding bone marrow biopsy. When the BM was considered as clonal (C or C/C), the abundance percentage and ratio of the clonotypes are also provided. When the pattern is scored polyclonal (irregular), but the index clonotypes of the lymphoma samples are represented in the polyclonal background, only the clonotype is noted in the table. No clonal overlap is reported when the index clonotype is not detected in the BM.

BL, Burkitt lymphoma; BM, bone marrow; bp, base pairs; C, clonal; DLBCL, diffuse large B‐cell lymphoma; FL, follicular lymphoma; MP, multiple products; MZL, marginal zone lymphoma; NA, not available; NSP, no specific product; P, polyclonal; PCB, polyclonal background; P irr, polyclonal irregular.

^a^ Expressed as a percentage of total B‐cells.

^b^ For the lymphoma cases, the clonal rearrangements are presented by their clonotype, the abundance percentage, and the ratio of the dominant #1 divided by an average measure of the background. When there is a dominance of two rearrangements, then the abundance and ratio of the #2 clonotype are also given.

**Figure 1:**
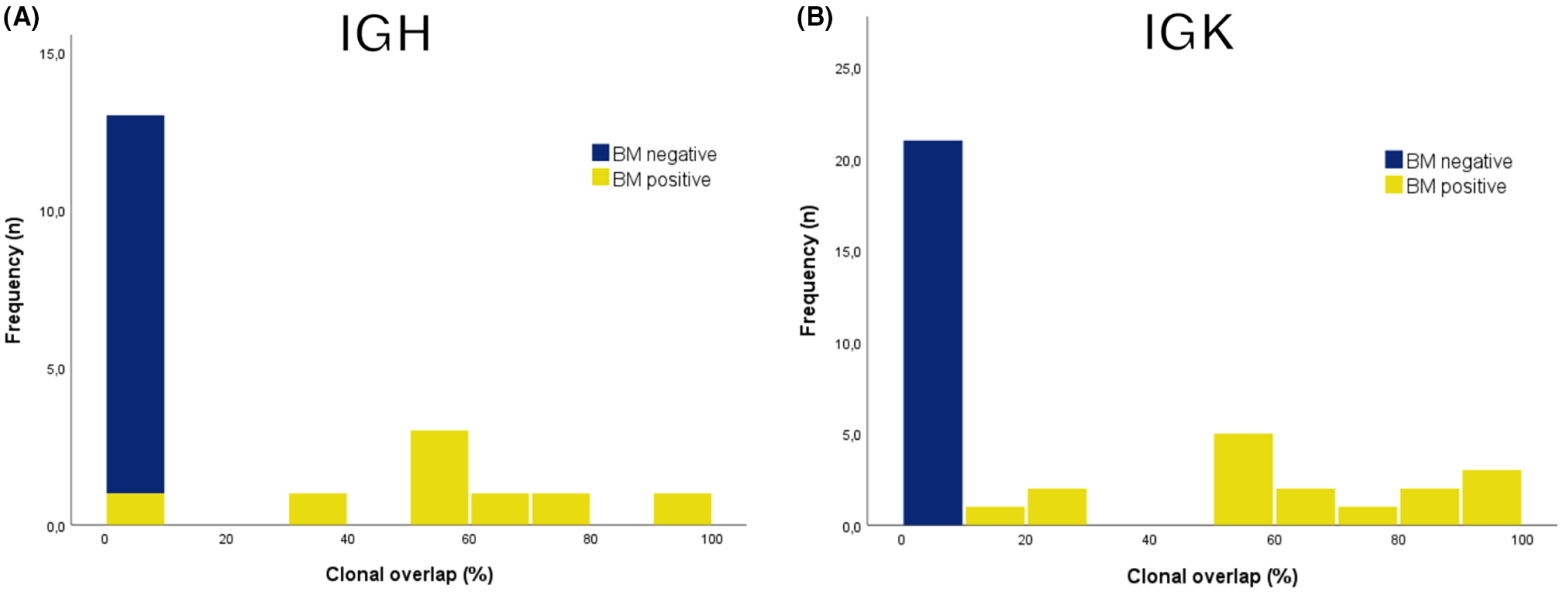
Distribution of clonal overlap for bone marrow biopsies positive (blue) and negative (yellow) for lymphoma by histology for IGH (**A**) and IGK (**B**) targets. The distributions show a clear distinction between bone marrow biopsies positive and negative for lymphoma, with high clonal overlap frequencies in bone marrow biopsies positive for lymphoma and low values for bone marrow biopsies without lymphoma.

As a measure for the presence of lymphoma infiltrate in the BM, the ‘clonal overlap’ was used. Clonal overlap was defined as the abundance percentage of primary lymphoma clonal rearrangements being present in the corresponding BM sample. As the immunoglobulin targets (1) IGHV‐IGHD‐IGHJ‐FR3; (2) IGHD‐IGHJ; (3) IGKV‐IGKJ and/or (4) IGKV‐KDE/IntronRSS‐KDE needed to be clonal in the primary lymphoma to allow their assessment in the BM, the number of targets that could be studied for clonal overlap differed per BM biopsy. A comparison of the clonal overlap on the four targets between the BM biopsies that were morphologically and immunologically positive (group 1, n = 11) and negative (group 2, n = 13) for lymphoma infiltrate showed a clear distinction (Figure 1), with an abundance percentage of 0%–1.9% of IGH targets and an abundance percentage of 0%–5.2% of IGK targets in 13 biopsies negative for lymphoma vs. 2.5%–93% abundance percentage (IGH targets) and 10%–97% abundance percentage (IGK targets) in eleven biopsies positive for lymphoma.

In BM biopsies from patients without lymphoma (n = 13), none of the evaluated IG targets including IGK‐DE was scored as clonal. The most frequently observed pattern in this category was ‘irregular polyclonal’ which reflects the limited number of B cells and the limited diversity in the BM (data not shown).

### NGS‐Based IG Clonality Analysis Can be of Diagnostic Help in Equivocal Bone Marrow Staging Biopsies

**Figure 2:**
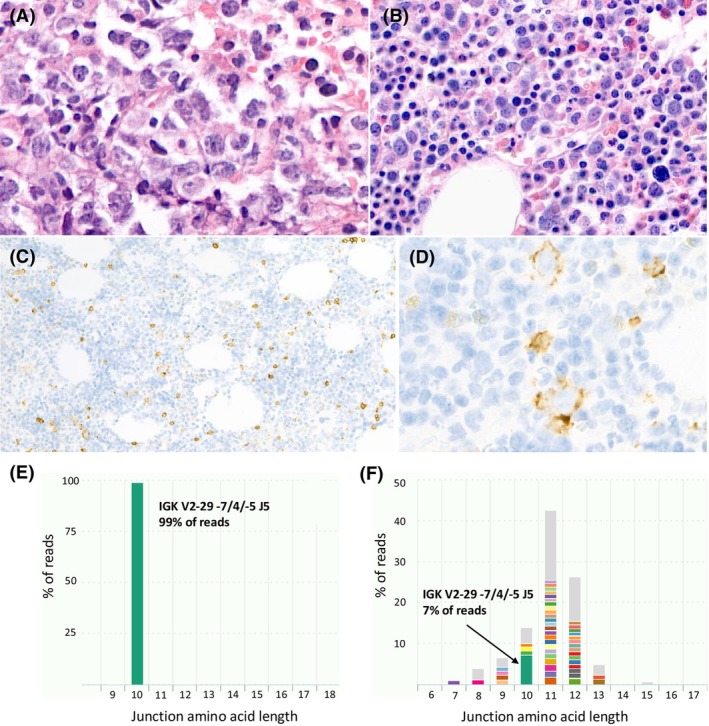
Subtle bone marrow involvement by lymphoma detected with IG NGS‐based clonality analysis (case no. 32). Based on a needle core biopsy of a supraclavicular lymph node (**A**), a diagnosis of diffuse large B‐cell lymphoma was made. A bone marrow staging biopsy showed a normocellular bone marrow without morphological evidence of lymphoma involvement (**B**). CD20 immunohistochemistry on the bone marrow biopsy showed a slight increase in small B‐cells with scattered larger cells (**C, D**). With NGS‐based IG clonality analysis, clonal products were detected for IGHV‐IGHD‐IGHJ, IGHD‐IGHJ, IGKV‐IGKJ, and Intron‐KDE. These clonal products were also detectable in the bone marrow with a frequency between 5 and 37% of reads, supporting bone marrow involvement by lymphoma. (**E, F**) show the NGS‐based clonality results for the IGKV‐IGKJ target for the lymph node (**E**) and the bone marrow (**F**). A clear clonal product is detected in the lymph node (green bar, 99% of reads). The same clonal product was detected in the bone marrow in 7% of reads (panel E/F green bar).

Subsequently, BM biopsies (group 3, n = 12) were studied in which differentiation between the presence or absence of lymphoma infiltrate could not be made with certainty. The number of informative targets varied between one and four targets (average 2.6, Table 3). Assessment of clonal overlap in these cases showed clearly different patterns between BM biopsies with very little clonal overlap (8 cases, clonal overlap 0–0.11%, IGH and IGK targets) and BM biopsies with a high amount of clonal overlap (4 cases, 4%–44%, IGH and IGK targets). Figure 2 represents an example of a case with a high amount of clonal overlap (case no. 32). Within one case, the different targets showed a high level of concordance, except for case no. 21 in which clonal overlap was 0% for IHGV‐IGHJ, but 44% for IGKV‐KDE/IntronRSS‐KDE, which is probably explained by the fact these are targets that do not undergo somatic hypermutation, whereas IGHV‐IGHJ does.

**Table 3:** NGS‐based IG clonality analysis results for n = 12 cases with equivocal bone marrow involvement (group 3)

Case no.	Lymphoma/bone marrow	DNA quality (bp)	B‐cell %	Neoplastic cell %^c^	NGS IG clonality analysis results							
					IGHV‐IGHJ FR3		IGHD‐IGHJ		IGKV‐IGKJ		IGKV‐KDE/IntronRSS‐KDE	
					Target score	Rearrangement(s): clonotype^d^	Target score	Rearrangement(s): clonotype^d^	Target score	Rearrangement(s): clonotype^d^	Target score	Rearrangement(s): clonotype^d^
1	MZL	200	70	90	C	*V3‐23(D)‐4/28/−7 J4 71%/41*	C + PCB	*D5‐12‐4/30/−5 J4 9%/3.0*	P	*NA*	P	*NA*
	BM	300	2	NA	P	*No clonal overlap*	P	*No clonal overlap*	P	*NA*	P	*NA*
2	MCL	400	70	90	C + PCB	*V4‐34‐3/13/−15 J6 36%/59*	P irr	*NA*	C + PCB	*V4‐1‐1/0/−0 J5 14%/26*	C/C	*V3(D)‐7–3/1/−0 Kde 15%/7.4* *intron − 0/4/−9 Kde 9%/4.6*
	BM	200	1	70	C + PCB	*V4‐34‐3/13/−15 J6 20%/7.4*	P irr	*NA*	P	*5% V4‐1‐1/0/−0 J5*	C/C	*V3(D)‐7–3/1/−0 Kde 14%/5.6* *intron − 0/4/−9 Kde7%/2.7*
3	MCL	300	90	90	C	*V3‐74–1/21/−3 J4 99%/2075*	NSP	*NA*	C/C	*V4‐1‐2/0/−2 J2 59%/433* *V2‐29–1/5/−3 J5 41%/299*	C	*intron − 0/0/−2 Kde 99%/1344*
	BM	200	2	5	P irr	*No clonal overlap*	P irr	*NA*	P irr	*No clonal overlap*	P irr	*No clonal overlap*
6	DLBCL	200	60	100	C/C	*V3‐74–1/21/−3 J4 64%/25* *V2‐5 = V2‐70‐0/30/−3 J4 22%/8.6*	NSP	*NA*	C	*V3(D)‐15–5/6/−2 J2 98%/339*	NSP	*NA*
	BM	300	1	NA	P irr	*No clonal overlap*	P irr	*NA*	P irr	*No clonal overlap*	MP	*NA*
12	DLBCL	300	50	90	P	*NA*	C	*D2‐8‐7/5/−11 J4 48%/58*	C/C	*V2‐29‐3/4/−0 J1 49%/446* *V3(D)‐15–0/2/−8 J5 34%/308*	C/C	*intron − 0/1/−8 Kde 55%/182* *intron − 0/0/−2 Kde 31%/101*
	BM	300	2	NA	P irr	*NA*	P	*D2‐8‐7/5/−11 J4 0.0032%*	P irr	*No clonal overlap*	P irr	*intron − 0/0/−2 Kde 0.0014%*
16	FL	200	50	80	NSP	*NA*	NSP	*NA*	MP	*NA*	C	*V1‐5‐2/0/−1 Kde 52%/9.2*
	BM	400	10	70	MP	*NA*	MP	*NA*	P irr	*NA*	P irr	*No clonal overlap*
19	MZL	400	80	70	C	*V4‐34‐0/38/−0 J5 93%/63*	C	*D1‐14‐4/10/−2 J4 96%/598*	C + PCB	*V2‐29–1/4/−4 J4 11%/7*	NSP	*NA*
	BM	300	5	50	P	*No clonal overlap*	P	*No clonal overlap*	P	*V2‐29–1/4/−4 J4 0.013%*	P	*NA*
21	DLBCL	400	60	80	C	*V3‐23(D)‐2/25/−6 J4 96%/341*	NSP	*NA*	NSP	*NA*	C/C	*V1(D)‐39–4/0/−7 Kde 52%/283* *V2‐24‐5/0/−2 Kde 47%/253*
	BM	300	5	50	MP	*No clonal overlap*	MP	*NA*	NSP	*NA*	C/C	*V1(D)‐37–5/0/−1 Kde 44%/10* *V2‐24‐5/0/−2 Kde 33%/7.4* *V1(D)‐39–4/0/−7 Kde* ^a^ *19%/4.9*
32	DLBCL	NA	90	90	C	*V4‐34‐0/44/−18 J6 99%/1086*	C	*D3‐22‐2/11/−5 J4 99%/676*	C	*V2‐29‐7/4/−5 J5 99%/1044*	C	*intron − 0/2/−9 Kde 99%/1229*
	BM	400	NA	NA	C + PCB	*V4‐34‐0/44/−18 J6 25%/13*	C + PCB	*D3‐22‐2/11/−5 J4 37%/15*	C + PCB	*V2‐29‐7/4/−5 J5 7%/4.9*	C + PCB	*intron − 0/2/−9 Kde 5%/1.7*
35	FL	200	80	80	C + PCB	*V3‐9‐0/41/−5 J4 49%/113*	P	*NA*	C/C	*V3(D)‐20–1/0/−1 J5 43%/213* *V4‐1‐2/0/−7 J3 25%/126*	C/C	*intron − 0/0/−3 Kde 46%/129* *intron − 3/4/−0 Kde 39%/112*
	BM	300	5	30	P	*No clonal overlap*	P	*NA*	P	*No clonal overlap*	P	*No clonal overlap*
37	BL	400	50	80	C	*V3‐15‐1/6/−15 J5 99%/788*	C	*D1‐26‐3/8/−7 J4 99%/1826*	C	*V2(D)‐28–4/0/−2 J4 99%/1522*	C/C	*intron − 3/1/−4 Kde 50%/372* *intron − 5/4/−6 Kde 48%/353*
	BM	400	5	20	C + PCB	*V3‐15‐1/6/−15 J5 19%/12*	C + PCB	*D1‐26‐3/8/−7 J4 14%/3.5*	C + PCB	*V2‐28 = V2D‐28‐4/0/−2 J4 4%/3.9*	C/C	*intron − 3/1/−4 Kde 17%/5.5* *intron − 5/4/−6 Kde 12%/3.9*
40	MZL	400	NA	NA	P	*NA*	C	*D2‐2‐6/6/−4 J5 30%/46*	P	*NA*	P	
	BM	400	NA	NA	P	*NA*	P	*D2‐2‐6/6/−4 J5 0.11%*	C + PCB	*V3(D)‐20–3/2/−10 J4 6%/6.8* ^b^	C + PCB	*V2(D)‐30–0/5/−10 Kde 13%/6.7* ^b^

For each case, NGS IG clonality analysis results are shown for the material on which the primary diagnosis of lymphoma was made and the corresponding bone marrow biopsy. When the BM was considered as clonal (C or C/C), the abundance percentage and ratio of the clonotypes are also provided. When the pattern is scored polyclonal (irregular), but the index clonotypes of the lymphoma samples are represented in the polyclonal background, only the clonotype is noted in the table. No clonal overlap is reported when the index clonotype is not detected in the BM.

BL, Burkitt lymphoma; BM, bone marrow; bp, base pairs; C, clonal; DLBCL, diffuse large B‐cell lymphoma; FL, follicular lymphoma; MCL, mantle cell lymphoma; MP, multiple products; MZL, marginal zone lymphoma; NA, not available; NSP, no specific product; P, polyclonal; PCB, polyclonal background; P irr, polyclonal irregular.

^a^ Three dominant clonotypes were observed for the BM sample of case 21, two of which overlap with the dominant clonotypes observed for the lymphoma sample. The third clonotype could not be found in the lymphoma sample. The protocol for amplification of the IGK targets was performed in duplicate for this case, yielding the same dominant clonotypes.

^b^ The clonal products in the IGK targets in the bone marrow biopsy in case 40 were not detected in the lymphoma and most likely correspond to a reactive process in the bone marrow.

^c^ Expressed as a percentage of total B‐cells.

^d^ For the lymphoma cases, the clonal rearrangements are presented by their clonotype, the abundance percentage, and the ratio of the dominant #1 divided by an average measure of the background. When there is a dominance of two rearrangements, then also the abundance and ratio of the #2 clonotype is given.

## Discussion

In this study, we applied NGS‐based IG clonality analysis to BM biopsies for lymphoma staging by detection of clonal IG gene rearrangements that were present in the primary lymphoma. We observed a clear distinction in the abundance of detected shared clonal IGHV‐IGHD‐IGHJ‐FR3, IGHD‐IGHJ, IGKV‐IGKJ and IGKintron/V‐IGKDE rearrangements between BM biopsies that were positive versus those that were negative for lymphoma based on morphology and immunohistochemistry. In BM biopsies negative for lymphoma, the index rearrangement might be detected but only at a low abundance percentage (0%–1.9% for IGH rearrangements and 0%–5.2% for IGK index rearrangements). In BM biopsies positive for lymphoma, the index rearrangements could be detected with an abundance of more than 2.5% for IGH and more than 10% for IGK.

In BM biopsies in which morphology and immunohistochemistry was insufficient to differentiate between the presence or absence of lymphoma, NGS‐based IG clonality analysis provided a binary outcome with a subset of cases with no or limited lymphoma derived IG gene rearrangements (<0.1% for both IGH and IGK) and a subset with clear presence of lymphoma derived IG gene rearrangements. Despite the small sample size, this study suggests that NGS‐based IG clonality analysis can be a valuable additional tool for difficult BM staging biopsies in patients with B‐cell lymphoma. Our results indicate that the NGS‐based clonality analysis for the assessment of BM involvement exhibits a substantial overall agreement with histopathological analysis with enhanced clonal detection rates over previously reported PCR‐based methods. Similar data have been presented by others, but with a different assay for larger amplicons and with a different study design.^10^, ^11^

Several studies have investigated BM involvement by clonality analysis and related this to prognosis.^7^, ^8^, ^9^, ^21^, ^22^, ^23^ As in this way also very subtle lymphoma infiltration without a morphological substrate might be detected, the question is whether this very subtle BM involvement has the same impact on prognosis and should have the same impact on treatment as morphologically defined BM involvement. Therefore, in our study, we took a different approach and focused on biopsies in which the pathologist was unsure whether there was BM involvement or not. This means that in the ‘equivocal’ category in our study (group 3), a morphological substrate was always present. As a diverse group of different lymphomas was selected for inclusion in this study, it was not possible to derive subtype‐specific conclusions for different forms of lymphoma.

Several aspects are of practical importance for using NGS‐based IG clonality analysis as described in this study. First, it is important to obtain DNA of sufficient quality from the BM specimen. Especially when no lymphoma‐related IG gene rearrangements are detected in the BM, it is important to assess that the DNA quality would have been sufficient to detect these rearrangements. At our institution, we apply a rapid protocol with EDTA calcification, which allows isolation of DNA of relatively high quality.^18^ For quality control, a size ladder PCR was performed. Notably, the EuroClonality‐NGS primer sets are expected to give amplicons of around 200 bp.

Second, it needs to be stressed that detection of lymphoma‐related IG gene rearrangements rather than looking for a clonal product per se is most relevant for defining BM involvement. This is illustrated by case no. 40 in which clonal rearrangements in a polyclonal background were detected for IGKV‐IGKJ and IGKV‐KDE/IntronRSS‐KDE in the BM, which was equivocal for lymphoma involvement based on morphology and immunohistochemistry. However, these IG gene rearrangements were not present as dominant clonotypes in the corresponding primary lymphoma sample. Moreover, the clonal IGHD‐IGHJ rearrangement that was detected in the lymphoma was only detected at a very low level in the BM. Probably, the dominant IG gene rearrangements detected in the BM reflect a passenger clone or a clone different from the lymphoma. Here, it is important to note that monoclonal B‐cell lymphocytosis (MBL) is a relatively common phenomenon, especially when using highly sensitive detection methods in older individuals.^24^ Bone marrow involvement in MBL is highly variable, ranging from no morphological changes to clear bone marrow involvement by MBL cells.^25^ It is vital not to confuse true bone marrow involvement by lymphoma with MBL and comparison of the clones will prevent this from happening.

If the clonality result in the lymphoma would not have been included in the analysis, this could have resulted in an erroneous diagnosis of BM lymphoma involvement. One caveat in using this approach of clonal overlap is that the BM could potentially contain a lymphoma that is different from the primary lymphoma. For evident lymphoma involvement based on morphology and immunohistochemistry, this will not be a diagnostic problem and it can be concluded that the patient has two separate lymphomas. For equivocal BM staging biopsies, it will be very difficult if not impossible to diagnose very subtle involvement of a second lymphoma or lymphoid premalignancy but it is expected that this has limited clinical relevance.

Third, the reliability of NGS‐based clonality analysis is dependent on the number of targets that can be studied. Especially the conclusion that there is no lymphoma involvement is more certain if all four targets are clonal in the lymphoma and can therefore be studied in the BM rather than basing the conclusion on only a single target. This is relevant especially for targets prone to somatic hypermutation (IGHV‐IGHD‐IGHJ, IGKV‐IGKJ) in which mutations in the primer binding site could result in the inability to detect a clonal rearrangement.

In conclusion, the detection of BM involvement in B‐cell lymphoma can be improved with the use of paired NGS‐based IG clonality analysis; the clearly recognizable subset of cases with high abundance of the clonal targets, and thus high clonal overlap suggests that NGS‐based IG clonality analysis can be used as additional information for evaluating whether a BM biopsy is positive or negative for lymphoma. When applying NGS‐based IG clonality analysis for this purpose, careful quality control assessment, comparison with the initial lymphoma and interpretation in the context of other parameters including morphology and immunohistochemistry results is vital.

## Author contributions

M.B. and P.J.T.A.G. designed the study. M.L., J.R. and J.A.C.W.L. performed N.G.S. analyses. M.B., M.L., A.W.L., K.M.H. and P.J.T.A.G. interpreted the results. M.L., M.B. and P.J.T.A.G. wrote the first version of the manuscript. All authors reviewed the manuscript.

## Funding

This study was supported by the Dutch Health Insurers' Innovation Fund (Project no. 17–179) and the revenues of the previously obtained patent (PCT/NL2003/000690), which is collectively owned by the EuroClonality/BIOMED‐2 Consortium and licensed to Invivoscribe.

## Conflicts of interest

The authors report no potential conflicts of interest.

## Data Availability

The data that support the findings of this study are available from the corresponding author upon reasonable request.
